# Influence of Manganese Content on Martensitic Transformation of Cu-Al-Mn-Ag Alloy

**DOI:** 10.3390/ma16175782

**Published:** 2023-08-24

**Authors:** Lovro Liverić, Tamara Holjevac Grgurić, Vilko Mandić, Robert Chulist

**Affiliations:** 1 Faculty of Engineering, University of Rijeka, Vukovarska 58, 51000 Rijeka, Croatia; lliveric@riteh.hr; 2 School of Medicine, Catholic University of Croatia, Ilica 242, 10000 Zagreb, Croatia; 3 Faculty of Chemical Engineering and Technology, University of Zagreb, Marulićev trg 19, 10000 Zagreb, Croatia; vmandic@fkit.hr; 4 Institute of Metallurgy and Materials Science, Polish Academy of Sciences, 25 Reymont Str., 30-059 Krakow, Poland; r.chulist@imim.pl

**Keywords:** Cu-Al-Mn-Ag alloys, shape memory alloy, heat treatment, microstructure, phase transformations, martensitic transformation, thermal analysis

## Abstract

The influence of manganese content on the formation of martensite structure and the final properties of a quaternary Cu-Al-Mn-Ag shape memory alloy (SMA) was investigated. Two alloys with designed compositions, Cu- 9%wt. Al- 16%wt. Mn- 2%wt. Ag and Cu- 9%wt. Al- 7%wt. Mn- 2%wt. Ag, were prepared in an electric arc furnace by melting of high-purity metals. As-cast and quenched microstructures were determined by optical microscopy and scanning electron microscopy equipped with EDS. Phases were confirmed by high-energy synchrotron radiation and electron backscatter diffractions. Austenite and martensite transformations were followed by differential scanning calorimetry and hardness was determined using the Vickers hardness test. It was found that the addition of silver contributes to the formation of the martensite structure in the Cu-Al-Mn-SMA. In the alloy with 7%wt. of manganese, stable martensite is formed even in the as-cast state without additional heat treatment, while the alloy with 16%wt. of manganese martensite transforms only after thermal stabilization and quenching. Two types of martensite, β_1_′ and γ_1_′, are confirmed in the Cu-9Al-7Mn-2Ag specimen. The as-cast SMA with 7%wt. Mn showed significantly lower martensite transformation temperatures, M_s_ and M_f_, in relation to the quenched alloy. With increasing manganese content, the M_s_ and M_f_ temperatures are shifted to higher values and the microhardness is lower.

## 1. Introduction

Shape memory alloys (SMAs) possess two unique effects, the memory effect and pseudoelasticity [1,2]. The most commercially used and also one of the most expensive SMAs is Nitinol (NiTi). However, currently it is increasingly being replaced in non-medical applications by Cu-based SMAs, which have excellent functional and electrical properties, low cost, and easy and cheap processability [3].

Both fundamental Cu-SMAs, those grounded in Cu-Zn and Cu-Al, are capable of undergoing a thermoelastic martensitic transition from the high-temperature β-parent phase, often referred to as the austenite phase, facilitated by rapid cooling or immersion in water for quenching. Under equilibrium conditions or slow cooling, the β-phase undergoes eutectoid decomposition to form a combined α + γ_1_-phase [4,5,6,7].

Cu-Al-Ni and Cu-Zn-Al are Cu-based SMAs and are the most commercially used SMAs, but they have limitations in some applications due to their high brittleness [8,9,10]. The addition of manganese to a Cu-Al alloy increases the ductility and cold workability of the material [11,12,13,14,15].

Previous research on Cu-Al-Mn SMAs has shown that the high-temperature β-phase, which is crucial for martensite formation, undergoes an order–disorder transformation, β (A2, BCC Cu) → β_2_ (B2, CuAl) → β_1_ (L2_1_, Cu2MnAl), during cooling [16,17,18]. 

The β-phase decomposes the eutectoid into α- and γ_2_-phases under equilibrium conditions. Similarly, the metastable martensite phase is formed from the β-phase by rapid cooling. Depending on the A2 or L2_1_ (Heusler) parent phase, the 2M or 6M martensite structure is formed [19]. Depending on the specific chemical composition, an array of martensite structures, including 2H (a hexagonal structure) and 9R, 18R, 6R, and 3R (rhombohedral structures), can manifest in Cu-based SMAs [20,21,22].

Thus far, the influence of some elements such as microalloying components on the functional properties of Cu-Al-Mn alloys has been studied. The results of microalloying with nickel showed that there is a decrease in grain size and an improvement in the shape memory effect, but at the same time there is a decrease in the ductility of the alloy and a shift in the martensitic transformation to lower values [23,24,25,26]. Moreover, the nickel is completely soluble in the β-phase, as are the microalloying elements Zn, Sn, and Au, forming a single-phase system, in contrast to Cu-Al-Mn microalloys with Fe, Ti, Cr, V, Co, and Si, which exhibit lower solubility in the matrix and a tendency to precipitate [25,27].

Moreover, microalloying of the Cu-Al-Mn SMA with Au, Co, and Zn does not increase the ductility of the material or the tendency of cold deformation, while the addition of Sn to the ternary alloy significantly reduces ductility [17]. Of the elements that are not completely soluble in the β-phase, Fe shows the greatest microalloying effect on the properties of Cu-Al-Mn alloys and, depending on the content added, increases the temperature of the martensitic transformation. Studies have also shown that the addition of Ti, Co, Cr, and Si mainly contributes to the poorer mechanical properties of Cu-Al-Mn SMAs [28,29,30]. Microalloying with magnesium, on the other hand, does not significantly change the properties of the ternary alloy and does not affect the change in martensitic transformation temperatures [18].

There are limited publications available on the effect of the addition of silver on the functional properties of Cu-Al-Mn SMAs. Silva [29] pointed out that the hardness, corrosion resistance, and ageing properties are improved by the addition of Ag to Cu-Al-Mn alloys. It was also found that silver increased the fraction of the ferromagnetic L2_1_-phase and the magnetic properties of the material, while Santos [30] reported an increase in the microhardness of the ternary alloy due to the formation of bainite [31].

The present work focuses on the effect of different compositions of a quaternary Cu-Al-Mn-Ag SMA on the formation of the martensite structure during casting and after thermal stabilization. The composition and microstructure were correlated with martensitic transformation temperatures and microhardness.

## 2. Materials and Methods

Cu-9Al-16Mn-2Ag and Cu-9Al-7Mn-2Ag alloys were prepared by melting raw metals (Mateck Material-Technologie & Kristalle, Jülich, Germany): copper 99.9%, aluminum 99.5%, manganese 99.8%, and silver 99.99%. The metals were melted in an electric arc furnace undergoing cycles of vacuuming and argon leaking and were re-melted four times for better homogenization.

The specimens were then cast in cylindrical molds with dimensions of 8 mm × 12 mm. Heat treatment was carried out at 900 °C for 30 min in chamber furnaces (OVER, Zagreb, Croatia) followed by quenching in water. 

For metallographic analysis, the samples were cut, cold mounted, and ground with 600#, 800#, and 1200# SiC abrasives followed by final polishing with 3 μm and 1 μm diamond paste performed on Citopress-20 and Tegramin-30 (Struers, Willich, Germany). The prepared specimens were etched with a 2.5 g FeCl_3_/48 mL CH_3_OH/10 mL H_2_O solution.

The microstructure was studied using an Axio Vert A1 optical microscope with the AxioCam ERc 5s microscope module (Carl Zeiss NTS GmbH, Oberkochen, Germany) and a scanning electron microscope (FEG QUANTA 250, FEI, Hillsboro, Oregon, USA) with an energy dispersion X-ray spectroscopy detector (EDS) (Oxford Instruments plc, Tubney Woods, Abingdon, Oxon, UK). 

Electron backscatter diffraction (EBSD) analyses were conducted using a Supra 35 scanning electron microscope (SEM) (Carl Zeiss NTS GmbH, Oberkochen, Germany) operating at an acceleration voltage of 15 kV, a working distance of 17 mm, a tilt angle of 70°, and step sizes ranging from 0.4 to 0.06 μm. Samples for EBSD analysis were prepared according to standard metallographic techniques, which included mechanical grinding using SiC papers, polishing with diamond pastes, and a final polishing step for 1 h using 0.04 μm colloidal silica.

The crystal structure and global texture of samples in the cast and quenched state were examined by high-energy X-ray diffraction measurements at DESY, Hamburg, Germany, using the beamline P07B (87.1 keV, λ = 0.0142342 nm). For phase analysis, the diffraction patterns were recorded in the so-called continuous mode using a 2D Mar345 Image Plate detector. In order to obtain textureless measurements, all samples were rotated by 180° about the ω-axis when X-rayed. To ensure the Bragg condition for all satellite reflections, the samples were continuously rotated around the ω sample axis by a ω < ±10°. The beam size was 1 × 1 mm^2^. Subsequently, the obtained 2D patterns were integrated using the Fit2D Version 18 (beta) software and presented in a graph of relative intensity vs. 2Theta angle. 

The atomic order was calculated as the intensity ratio of I_hkl_/I_220_ using the reflection of the dominant phase, i.e., up to 900 °C from austenite reflections, and then from martensite reflections.

Transformation temperatures were determined using a Modulated Differential Scanning Calorimeter (MDSC) Mettler-Toledo 822e (Mettler-Toledo, Columbus, OH, USA). Dynamic measurements were performed by 2 heating/cooling measurement cycles from −100 °C to 350 °C in an inert atmosphere with a heating/cooling rate of 10 K/min. 

The microhardness of the alloys was determined using the Future Tech FM-ARS-F-9000 with an FM-700 microhardness tester (FM-ARS 9000, Future-Tech, Kanagawa, Japan) using a load of HV 100 g and a dwell time of 15 s. The Vickers microhardness values were calculated as the average of five individual measurements taken from each sample.

## 3. Results

**Figure 1:**
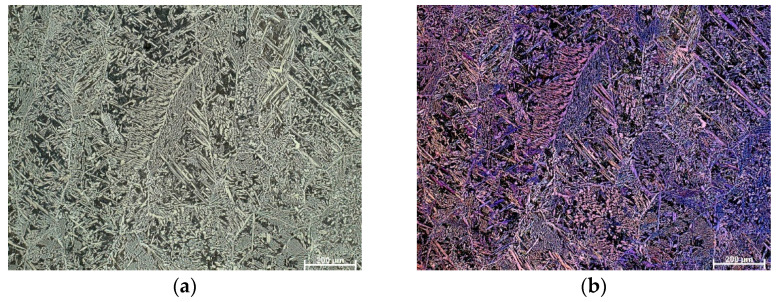
OM micrographs of as-cast Cu-9Al-16Mn-2Ag SMA: (**a**) BF, mag. 100×, (**b**) POL, mag 100×.

**Figure 2:**
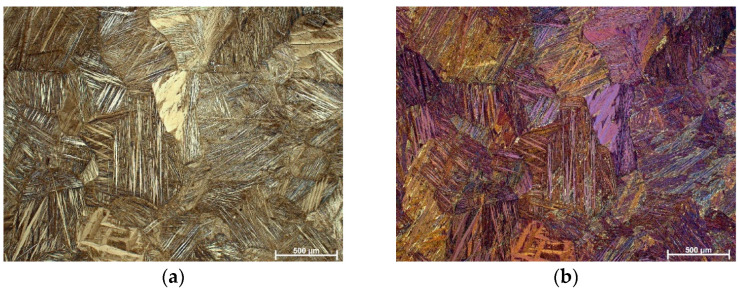
OM micrographs of quenched Cu-9Al-16Mn-2Ag SMA: (**a**) BF, mag. 50×, (**b**) POL, mag 50×.

OM micrographs in the bright field and polarized light of the as-cast Cu-9Al-16Mn-2Ag SMA are shown in Figure 1. A two-phase morphology, (α + β), is observed in the microstructure, with some very thin needles of martensite forming at the grain boundaries. After solution treatment and quenching, the morphology changed, and a completely formed martensite structure with different orientations inside grains was observed (Figure 2). The grain size in the quenched Cu-9Al-16Mn-2Ag alloy is significantly smaller. The grain size is influenced by parameters of thermal stabilization, i.e., temperature, retention time, cooling medium, etc. [32]. In Cu-Al-Mn alloys during the heat-induced thermoelastic martensite transformation, two martensite structures, β_1_′ (18R) and γ_1_′ (2H), can co-exist, depending on chemical composition, the e/a ratio, and thermal stabilization routes [33].

Increasing structural order stabilizes more γ_1_′-martensite than β_1_′-martensite, while the driving force for the nucleation of martensite sites is higher for the 2H-type martensite. The two types of martensites are very similar; they only show differences in morphology caused by different modes of inhomogeneous shear. The β_1_′ (18R)-martensite is formed from the L2_1_ (Heusler) phase, and its stacking sequence is AB’CB’CA’CA’BA’BC’BC’AC’AB’. The γ_1_’-martensite is usually formed at higher aluminum compositions, specifically more than 13 at%.

**Figure 3:**
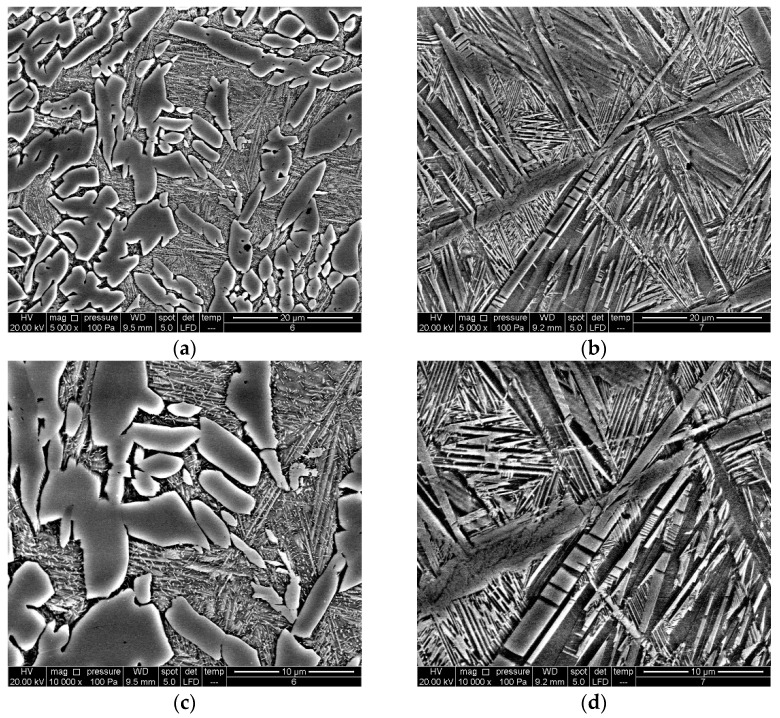
SEM micrograph of Cu-9Al-16Mn-2Ag SMA, mag. 5000×: (**a**) as-cast state, (**b**) quenched state, mag. 10,000×, (**c**) as-cast state, (**d**) quenched state.

The SEM analysis of the investigated samples confirmed the complete transformation from austenitic to martensitic structure after quenching without the precipitation of the α-phase (Figure 3b,d). The mostly spear-like shape of martensite can be observed in the quenched alloy, which refers to the β_1_′-type of martensite, with a monoclinic structure. At some parts a zig-zag morphology of the β_1_′-martensite is also detected (Figure 3d). Some coarse shape martensite plates can be also observed in the quenched alloy with 16%wt. of manganese (Figure 3d). Most likely, this can point to the existence of small amounts of another martensite type, γ_1_′, with an orthorhombic structure, but it should be confirmed by XRD analysis. The martensite pattern depends on the nucleation process and growth-type kinetics. It is well known that during thermoelastic martensitic transformation the growth of martensite plates takes place progressively and the formation of new plates occurs only when existing plates cannot grow further due to grain boundaries [33]. Needles of the β_1_′-martensite type exhibit high thermoelastic behavior attributed to controlled growth in self-accommodating groups. 

Martensite plates nucleate and grow at different sites, as can be observed in the SEM micrographs (Figure 3). The SEM micrographs reveal the initial stages of needle formation between α-precipitates in the as-cast alloy (Figure 3a,c). 

**Figure 4:**
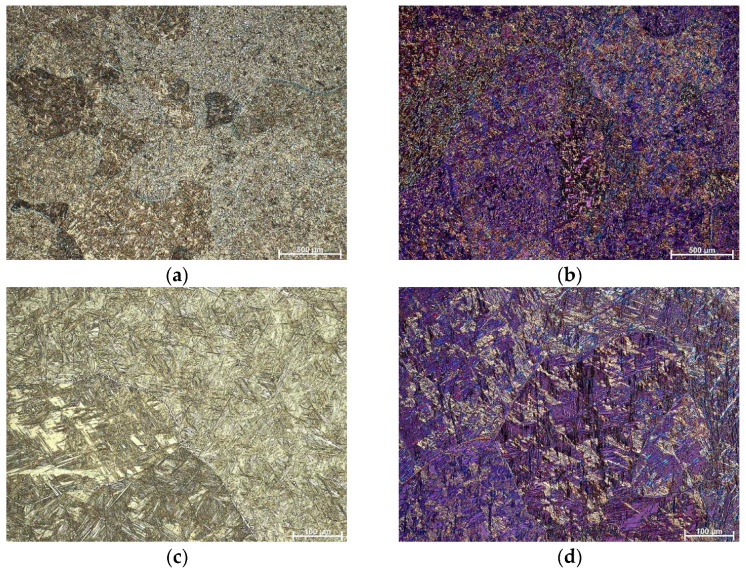
OM micrographs of as-cast Cu-9Al-7Mn-2Ag SMA: (**a**) BF, mag. 50×, (**b**) POL, mag 50×, (**c**) BF, mag. 200×, (**d**) POL, mag. 200×.

**Figure 5:**
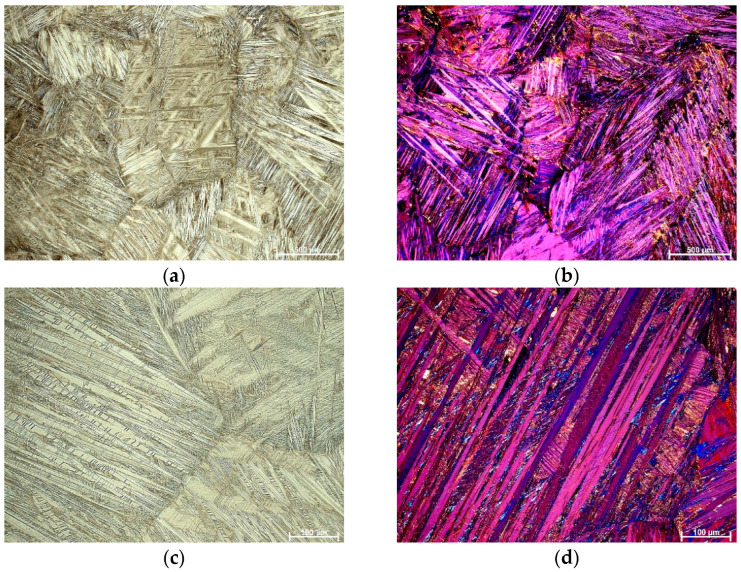
OM micrographs of quenched Cu-9Al-7Mn-2Ag SMA: (**a**) BF, mag. 50×, (**b**) POL, mag 50×, (**c**) BF, mag. 200×, (**d**) POL, mag. 200×.

**Figure 6:**
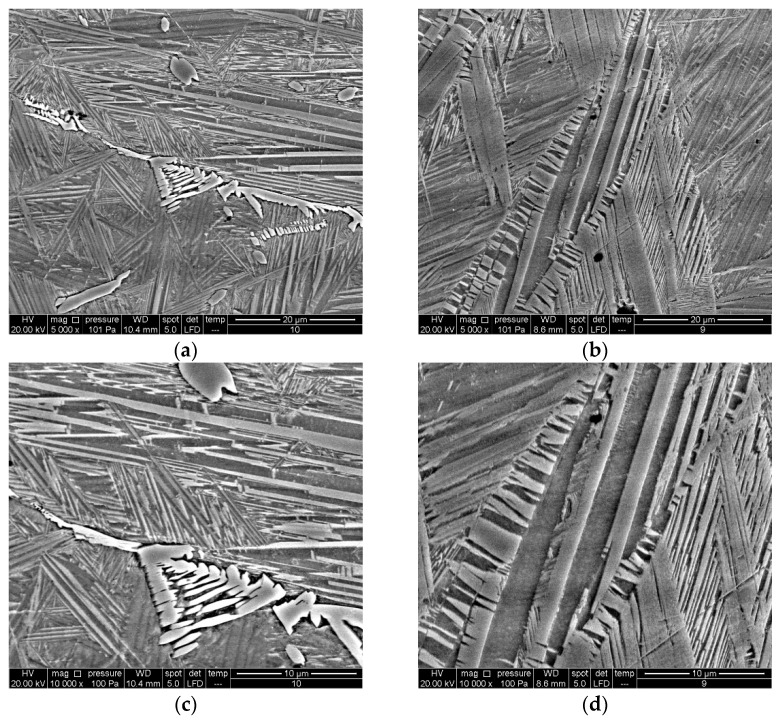
SEM micrograph of Cu-9Al-7Mn-2Ag SMA, mag. 5000×: (**a**) as-cast state, (**b**) quenched state, mag. 10,000×, (**c**) as-cast state, (**d**) quenched state.

**Figure 7:**
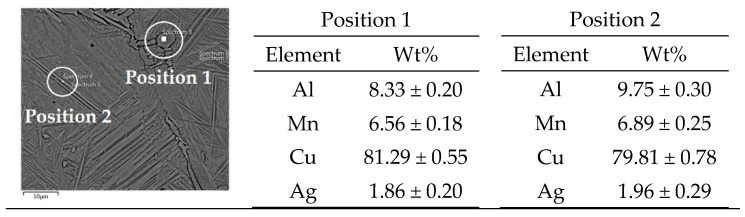
EDS analysis of as-cast Cu-9Al-7Mn-2Ag alloy.

**Figure 8:**
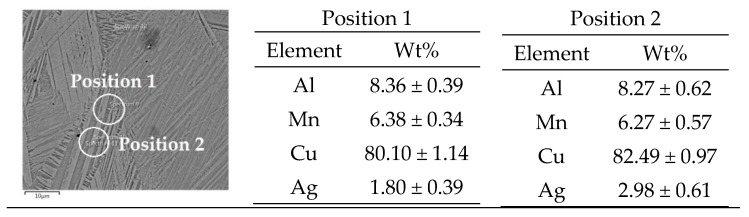
EDS analysis of quenched Cu-9Al-7Mn-2Ag alloy.

Figure 4 and Figure 5 display the fully formed martensite structure in the alloy with lower manganese content in both the as-cast and quenched states. The quenched alloy exhibits more intense and thicker martensite formed by twinning, as depicted in Figure 6. The EDS analysis reveals a similar composition in various positions of the martensite matrix for both the as-cast (Figure 7) and quenched (Figure 8) states of the Cu-9Al-7Mn-2Ag alloy.

**Figure 9:**
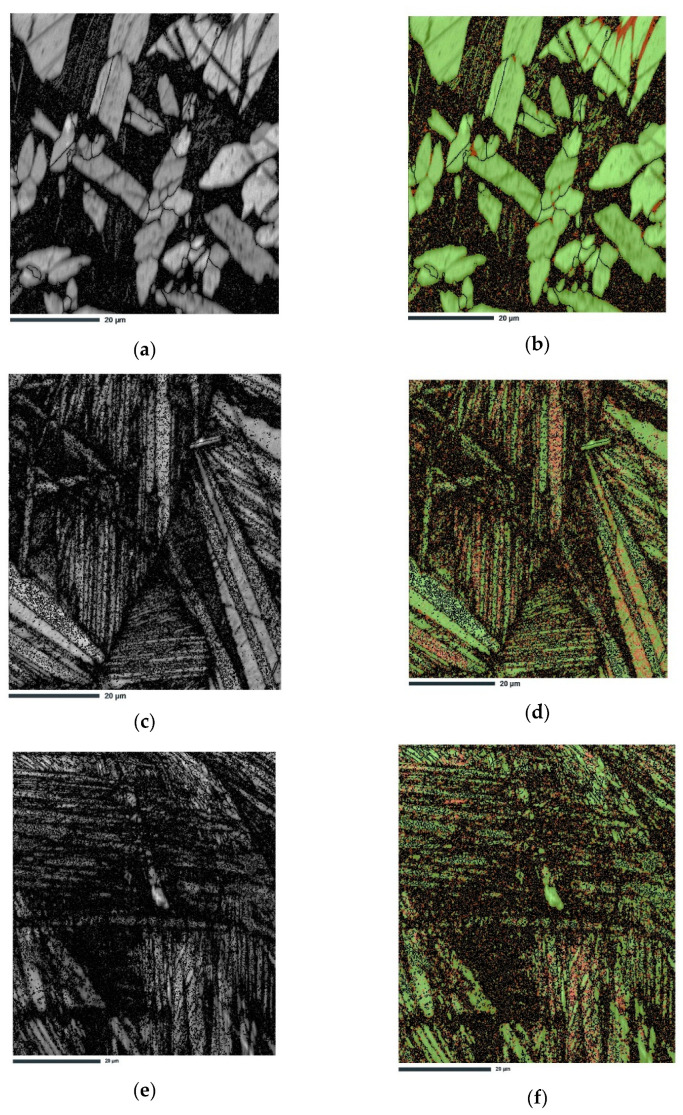
Results of EBSD measurements: (**a**) band contrast image, (**b**) phase map for Cu9Al16Mn2Ag alloy in the as-cast state; (**c**) band contrast image, (**d**) phase map for Cu9Al16Mn2Ag alloy in the quenched state; (**e**) band contrast image, (**f**) phase map for the Cu9Al7Mn2Ag alloy in the quenched state.

Figure 9 shows the BS images, band contrast, and phase maps for the Cu-9Al-16Mn-2Ag alloy in the as-cast state, with the precipitates of the fcc-Cu-phase (α-phase) colored green. Figure 9e,f primarily depicts spear-shaped morphologies in the β_1_’-martensite plate group in the quenched alloy. The EBSD analysis reveals varying types of patterns in microareas associated with habit variants of the 18R martensite.

**Figure 10:**
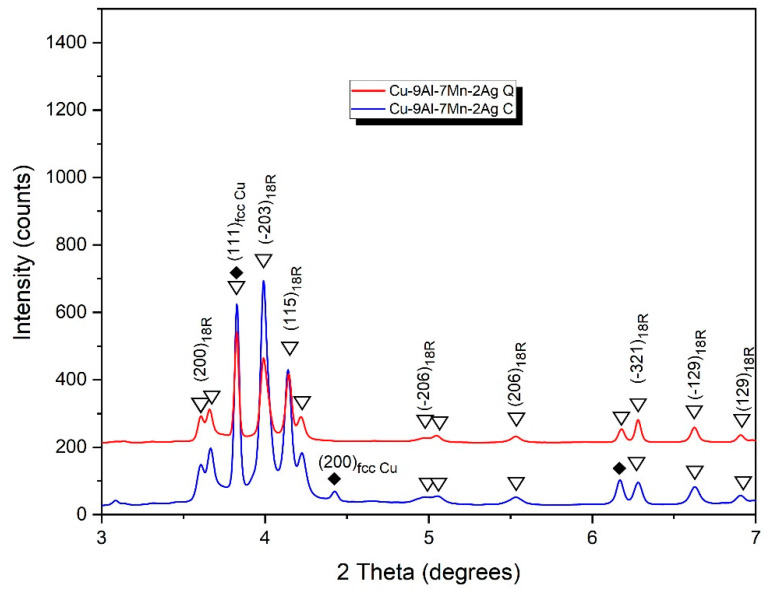
XRD diffractogram for Cu-9Al-7Mn-2Ag alloy.

**Figure 11:**
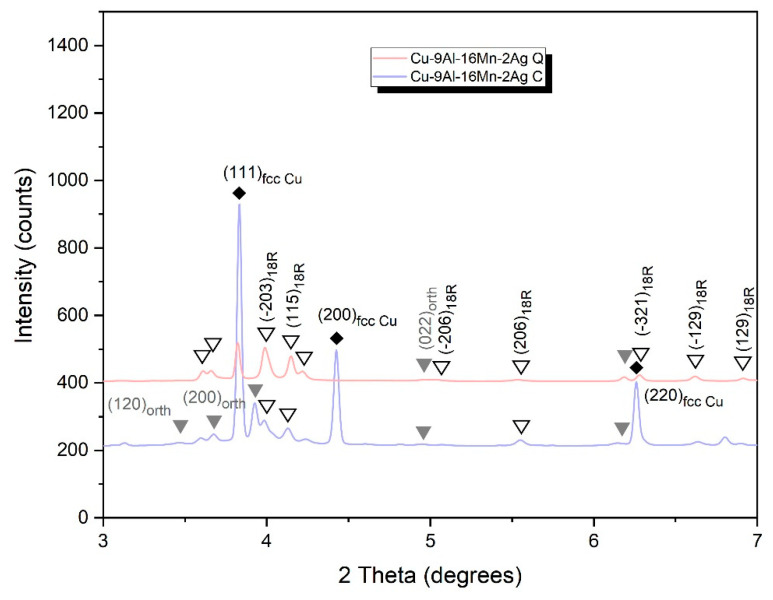
XRD diffractogram for Cu-9Al-16Mn-2Ag alloy.

The XRD analysis confirmed the existence of 18R (β_1_’)-martensite in both the as-cast and quenched Cu-9Al-7Mn-2Ag alloy (Figure 10). In the as-cast alloy with 16 wt.% of manganese, intensive peaks for fcc Cu (α-phase) are detectable, indicating that a very low cooling rate gives rise to Cu precipitation. On the other hand, quenching the alloy leads to the formation of 18R-martensite, similar to that observed in the Cu-9Al-7Mn-2Ag SMA (Figure 11). The XRD diffractogram for the Cu-9Al-16Mn-2Ag alloy confirmed the co-existence of two martensitic phases, β_1_′ (18R) and orthorhombic γ_1_′ (2H).

**Figure 12:**
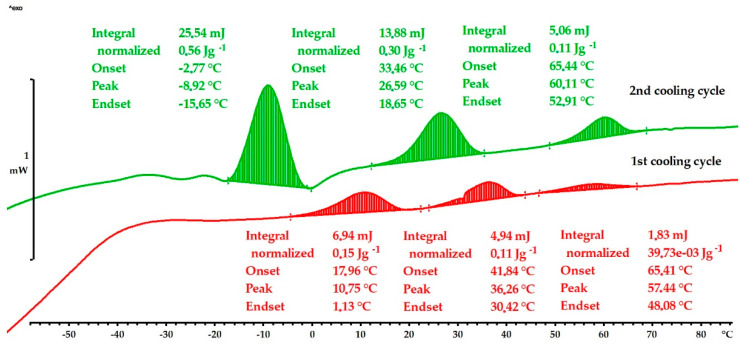
DSC cooling curves for quenched Cu-9Al-16Mn-2Ag alloy.

**Figure 13:**
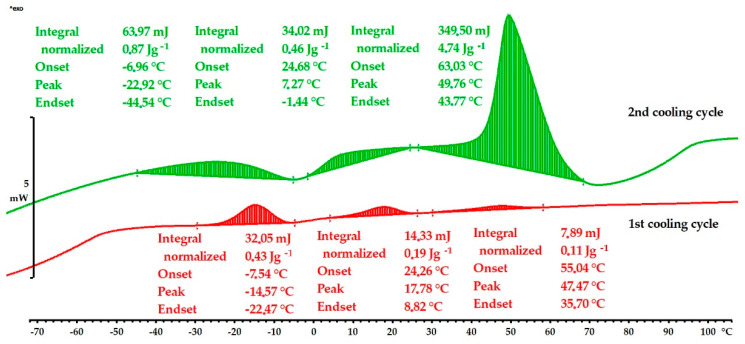
DSC cooling curves for quenched Cu-9Al-7Mn-2Ag alloy.

The DSC results and transitions are presented in Table 1 and in Figure 12 and Figure 13. A martensite transformation is a first-order transition, and it is not solely related to the change in specific heat capacity (cp) but is also accompanied by the emission of fusion heat during the transformation. The martensite transformation in the quenched Cu-9Al-16Mn-2Ag alloy shows that the start of the martensitic transformation was at M_s_ = 65 °C in both cooling cycles, and the finish temperatures were at M_f_ = 1 °C (1st cooling cycle) and M_f_ = −15 °C (2nd cooling cycle), respectively (Figure 12). With a lower content of manganese, 7 wt.%, the as-cast sample shows a significantly lower martensitic start temperature, M_s_ = 22 °C, in the first cooling cycle, and M_f_ = −56 °C (Table 1). After quenching, transition temperatures were shifted to higher values, M_s_ = 55 °C and M_f_ = −22 °C (1st cycle) and M_s_ = 63 °C and M_f_ = −44 °C (2nd cycle). Quenched samples with 7 wt.% and 16 wt.% of manganese exhibited similar martensitic transformation temperatures, but the enthalpy of transformation was significantly higher in the Cu-9Al-7Mn-2Ag alloy due to the more intense formation of martensite layers (Figure 13). The multiple exothermic peaks observable in the DSC thermograms are related to re-orientations and different martensitic structures.

The microhardness of the studied Cu-Al-Mn-Ag shape memory alloy (SMA) is presented in Table 2. The quenched Cu-9Al-7Mn-2Ag alloy exhibits slightly lower microhardness compared to the as-cast condition, a finding that aligns with previous SMA investigations [16,29,30,31,34,35]. Increasing the manganese content to 16 wt.% results in a decrease in the hardness of the Cu-SMA. In contrast to the Cu-9Al-7Mn-2Ag alloy, the Cu-SMA with 16 wt.% manganese displays higher microhardness in the quenched state, which is atypical for shape memory materials and could be linked to the formation of the brittle γ_1_’-phase in the quenched alloy.

According to Silva [29], the addition of Mn to the Cu-11%Al alloy significantly increases its microhardness value, while the addition of 3% Ag leads to a slight decrease in the microhardness of the Cu-11%Al alloy. Silva [29] also notes that Mn alters the range of phase stability and that the eutectoid reaction is no longer detectable in the annealed Cu-11%Al and Cu-11%Al-3%Ag alloys. The presence of Ag does not significantly influence the phase transformation sequence or microhardness but increases the magnetic moment of the Cu-11%Al-10%Mn alloy by about 2.7 times and decreases the rates of eutectoid and peritectoid reactions in the annealed Cu-11%Al alloy.

Furthermore, according to Jain [16], increasing the Al:Mn ratio in as-cast samples of the alloy system leads to an increase in hardness. However, in quenched samples, the hardness decreases, and this decrease is largely consistent with the Al:Mn ratio, which is attributed to the formation of softer martensitic phases at higher ratios. 

## 4. Conclusions

In this work, we investigated the microstructures and phase transitions of Cu- 9wt.% Al- 16wt.% Mn- 2wt.% Ag and Cu- 9wt.% Al- 7wt.% Mn- 2wt.% Ag shape memory alloys (SMA), examining the differences between the as-cast and quenched states. We found that the Cu- 9wt.% Al- 7wt.% Mn- 2wt.% Ag SMA exhibits a stable martensite morphology in both the as-cast and quenched states, while the Cu- 9wt.% Al- 16wt.% Mn- 2wt.% Ag SMA develops a martensitic structure only after heat treatment and quenching in water. In both Cu alloys, the existence of 18R (β_1_’)-martensite is confirmed, but the γ_1_’-phase is present only in the Cu-9Al-16Mn-2Ag alloy. The Ms temperatures for the quenched samples are notably similar, at 63 °C or 65 °C, but the Mf value shifts to lower values at a manganese content of 7 wt%. Furthermore, our study shows that increasing the manganese content leads to a decrease in microhardness, with the quenched Cu-9Al-7Mn-2Ag alloy exhibiting lower microhardness compared to its as-cast counterpart.

## Figures and Tables

**Table 1:** DSC results of martensitic transformation temperatures and fusion enthalpy.

Sample	M_s_/°C (1st Cycle)	M_f_/°C (1st Cycle)	M_s_/°C (2nd Cycle)	M_f_/°C (2nd Cycle)	ΔH (J/g)
as-cast Cu-9Al-7Mn-2Ag	22	−56	14	−63	2.25
quenched Cu-9Al-7Mn-2Ag	54	−22	64	−44	6.8
quenched Cu-9Al-16Mn-2Ag	65	1	65	−15	1.27

**Table 2:** Microhardness of Cu–Al–Mn-Ag SMAs.

Hardness		HV					
		1	2	3	4	5	Average
Cu9Al16Mn2Ag	As-cast state	167.27	192.51	201.81	170.12	180.97	182.54
	Quenched state	244.93	235.50	237.01	238.24	241.69	239.47
Cu9Al7Mn2Ag	As-cast state	241.72	248.14	246.48	244.68	246.12	245.42
	Quenched state	241.72	248.14	238.52	243.67	239.84	242.38

## Data Availability

Not applicable.
